# SARS-CoV-2 Infection among the Dental Staff from Lombardy Region, Italy

**DOI:** 10.3390/ijerph18073711

**Published:** 2021-04-02

**Authors:** Silvano Gallus, Luca Paroni, Dino Re, Riccardo Aiuto, Davide Maria Battaglia, Rolando Crippa, Nicolò Carugo, Matteo Beretta, Lorenzo Balsano, Luigi Paglia

**Affiliations:** 1 Department of Environmental Health Sciences, Istituto di Ricerche Farmacologiche Mario Negri IRCCS, 20156 Milan, Italy; silvano.gallus@marionegri.it (S.G.); luca.paroni@marionegri.it (L.P.); 2 Department of Biomedical, Surgical, and Dental Science, University of Milan, 20122 Milan, Italy; dino.re@unimi.it (D.R.); riccardo.aiuto@alice.it (R.A.); battagliadavidemaria@gmail.com (D.M.B.); 3 Fondazione Istituto Stomatologico Italiano, 20122 Milan, Italy; rolcrip@icloud.com (R.C.); n.carugo@gmail.com (N.C.); 4 Private Practice in Orthodontics and Paediatric Dentistry, 21100 Varese, Italy; teoberet@libero.it; 5 Private Practice in Dentistry, Pandino, 26065 Cremona, Italy; l.balsano@studiobalsano.it

**Keywords:** Sars-Cov-2, coronavirus, COVID-19, dentists, antibody, Italy

## Abstract

Dentists have been supposed to be among the healthcare workers at greatest risk of SARS-CoV-2 infection. However, scant data are available on the issue. The aim of this study is to quantify the SARS-CoV-2 antibody prevalence and determinants in a sample of dentists, dental hygienists, and other personnel employed among the dental staff in Lombardy region. We used an accurate rapid diagnostic test kit detecting immunoglobulins (Ig) in 504 adults. Of the 499 participants who obtained a valid antibody test, 54 (10.8%) had a SARS-CoV-2 positive test (0.4% IgM+, 1.8% both IgM+ and IgG+, and 8.6% IgG+). A statistically significant association with infection was found for geographic area (compared to Milan, adjusted odds ratio was 2.79, 95% confidence interval, CI: 1.01–7.68 for eastern and 2.82, 95% CI: 1.34–5.94, for southern Lombardy). The clinical staff did not result positive to SARS-CoV-2 more frequently than the administrative staff. This is the first study using antibody test in the dental staff personnel. It shows that the prevalence of SARS-CoV-2 infection in Lombardy region was around 10%, in line with estimates on other healthcare professionals. Despite the close physical contact with the patient, dentists have been able to scrupulously manage and effectively use protective devices.

## 1. Introduction

In December 2019, a new coronavirus named SARS-CoV-2 was reported to the WHO Country Office in China. Although SARS-CoV-2 is asymptomatic for the majority of infected people, this coronavirus can cause a respiratory disease, named COVID-19, progressing in some cases to atypical bronchial pneumonia not responding to treatment. COVID-19 is lethal for approximately 10% of symptomatic subjects, the death rate being higher in men, older subjects, and people with concomitant chronic conditions. From the Wuhan region of China, the virus spread globally. Italy was the first country where the outbreak spread outside Asia. SARS-CoV-2 was first detected on 21 February 2021, but was present in the Lombardy region weeks before the first official case was confirmed [1]. In Lombardy, the richest Italian region with the highest number of international trades, the largest number of residents (over 10 million), and the highest population density, SARS-CoV-2 substantially spread, particularly in eastern provinces. Lombardy remains today among the most hit by COVID-19 areas worldwide. COVID-19 has killed almost 20,000 people in Lombardy and infected more than 400,000 [2], by far the highest rate in Italy [3,4]. 

To detect the virus SARS-CoV-2, laboratories globally use nucleic acid amplification tests (NAATs) mainly based on reverse transcription polymerase chain reaction (RT-PCR) assays [5,6]. Although these tests are highly accurate [7], limited access, capacity limitations, and associated costs led to the development of fast and cheap rapid diagnostic tests (RDTs) to diagnose SARS-CoV-2. RDTs can detect either antigens (Ag) or antibodies (Ab) and are able to provide a response in 15 to 40 min [8]. Ag-RDTs directly detect the presence of the virus indicating a current virus replication and therefore an active infection. Ab-RDTs detect immunoglobulins (Ig) IgM and IgG or a combination of them. Immunoglobulins are produced during an active infection but are also detectable after the virus has been eradicated, indicating therefore a previous infection [9]. IgM and IgG can be detected even after 48 days from disease onset symptoms [10,11,12]. In particular, the response of the immunosystem is first associated with an increased level of IgM while followed by an increase of IgG [13]. For the ease of performing the test and the speed in providing a response, Ab-RDT are best used in surveillance systems to guide public health measures and to quantify seroprevalence at a population level [8].

Healthcare providers, being at the frontline of response to COVID-19, are considered a population at high risk of acquiring the disease. A systematic review aimed at quantifying the prevalence of SARS-CoV-2 infection among healthcare workers found 46 studies assessing infection through RT-PCR, showing a pooled prevalence estimate of 11% overall, 19% among symptomatic subjects, 8% among both symptomatic and asymptomatic, and 5% among asymptomatic workers [14]. The same review also identified 28 studies evaluating prevalence of antibodies against SARS-CoV-2, showing a pooled infection prevalence of 7% [14]. The estimates substantially varied according to country and type of personnel [14]. A study based on a sample of 3985 healthcare workers located in seven different hospitals across Lombardy region found a higher IgG positive prevalence (i.e., 13%) [15], compared to the global pooled estimate [14]. Among healthcare providers, dentists, dental hygienists, and support personnel are considered a particularly high-risk category of getting infected as they perform their daily activity in close contact with patients’ aerosol and droplets form oral cavities [16,17].

Despite the potentially high risk to get infected with SARS-CoV-2 among dentists, the prevalence of SARS-CoV-2 positive subjects among dentists detected through RT-PCR diagnostics was 0.8% in China and 0.9% in the US [18,19]. In a descriptive quantitative study among dentists in Spain, prevalence of SARS-CoV-2 positive subjects was 1.9% in April 2020, 3.0% in June 2020, and 1.3% in September 2020 [20]. Preventive measures and protocols already in use among dentists even before COVID-19 pandemic could have had a favorable role in limiting the spread of SARS-CoV-2 among dentists [21]. To our knowledge, no study on dentists has been conducted so far to evaluate the spread of SARS-CoV-2 infection using Ab-RDTs.

The aim of this study is to quantify the SARS-CoV-2 antibody prevalence and determinants of a sample of dentists, dental hygienist, and other personnel working in dental setting from Lombardy region, where the prevalence of SARS-CoV-2 infection in the general population has been found to be relatively high—approximately 5–11% [22,23], with selected areas showing even higher prevalence of infection—up to 39% in Bergamo [24].

## 2. Materials and Methods

An observational study was conducted on a sample of administrative and dental staff employees from Lombardy region who volunteered to be tested through an Ab-RDT for the detection of COVID-19. The study has been conducted from 28 May 2020 up to 30 September 2020 after having obtained the approval from the ethics committee of Università Statale di Milano, Milan (n: 61/20). For the preparation of the present analysis, we followed the STROBE guidelines for cross-sectional studies [25].

In order to be eligible for the study, subjects had to be 18 years or older and be part of the following working categories: dentists, dental technicians, resident dental doctors, dental hygienists, prosthodontic students, dental hygiene students, dental office assistants, nurses, laboratory technicians, administrative, secretaries, managers working in dental public or private institute or dental clinical centers located in Lombardy. Subjects who did not work in dental setting or refused to participate to the study were excluded from the study. Volunteers were recruited through mailing list and social networks.

By protocol, we aimed to reach a sample of 500 subjects. With such a sample size, assuming a 10% prevalence of SARS-CoV-2 positive subjects, we are able to estimate the prevalence of positive subject with a standard error (SE) lower than 1.4%, leading to a 95% confidence interval (CI) of +/− 2.6% with a statistical power of 80% and a probability of type-I error (α) of 5%. Therefore, the first 500 volunteers accepting to participate were enrolled in the study.

An immunochromatography test KHB^®^ Diagnostic Kit (KHB, Shanghai, China) for SARS-CoV-2 IgM/IgG (ab-RDT-KHB) was provided to each participant to verify the serological status of the subjects. The ab-RDT-KHB detects the presence or absence of IgM and IgG against SARS-CoV-2 in either serum, plasma, or whole blood, providing a visible line on the diagnostic kit cassette. In case of using whole blood, a sample of 20 µL is used and the diagnostic result is obtained in 15 min [26]. ab-RDT-KHB sensitivity, estimated on an Italian population sample of 135 subjects for tests performed 16 days from the first symptoms, and ab-RDT-KHB specificity, estimated on an Italian population sample of 272 subjects, are respectively, 95.1% and 99.3% [27].

Each study participant also completed an on-line questionnaire in an ad hoc developed website. The questionnaire included information on socio-demographic characteristics (age, sex, and municipality of residence), anthropometric variables (self-reported height and weight), COVID-related information (influenza-like symptoms in the previous 3–4 months, personal perception of being SARS-CoV-2 positive subject, and SARS-CoV-2 positive subjects among family members), occupation (professional role and work during COVID-19 lockdown), and selected lifestyle habits (cigarette smoking, use of heated tobacco products, HTP, electronic cigarette use, and daily consumption of different types of alcoholic beverages). A few missing values were present for height and weight, municipality of residence, electronic cigarette, and HTP use.

Study participants were categorized in the following four geographic areas: province of Milan, northern Lombardy (provinces of Como, Lecco, Monza-Brianza, Sondrio, and Varese), and eastern (provinces of Bergamo and Brescia) and southern Lombardy (Cremona, Lodi, Mantova, and Pavia). We categorized participants according to the size of their municipalities: large municipalities (i.e., capitals of provinces) or small municipalities (other municipalities). We computed body mass index (BMI) as the ratio between weight (kg) and height (m^2^). BMI was categorized into three groups, normal weight (BMI < 25.0 kg/m^2^), overweight (BMI 25.0–29.9 kg/m^2^), and obesity (BMI ≥ 30.0 kg/m^2^).

### Statistical Analysis

We present descriptive analyses, including the percent prevalence of the population according to SARS-CoV-2 test, overall and by sex and age group. To quantify the relationship between potential risk factors and COVID-19, we derived the adjusted odds ratios (AOR) of being tested positive to SARS-CoV-2, and corresponding 95% confidence intervals (CI), using multiple logistic regression models after adjustment for age and sex. All the analyses were performed in SAS 9.4 (SAS Institute, Cary, NC, USA).

## 3. Results

Overall, 504 workers among the dental staff in Lombardy were enrolled in this study. Of these, five participants (1.0%; one man and four women) did not obtain a valid test and were excluded from all the analyses. Of the 499 participants who obtained a valid antibody test, 67% were women and 33% were men, mean age was 43.9 years (SD: 14.4) overall, 41.4 (SD: 13.0) among women, and 48.7 years (SD: 15.7) among men. In all, 445 (89.2%) had a negative and 54 (10.8%) a positive test (0.4% IgM+, 1.8% both IgM+ and IgG+ and 8.6% IgG+, Table 1).

Table 2 shows the multivariate AORs of being positive to the antibody test according to selected socio-demographic characteristics. Compared to subjects resident in the province of Milan, those resident in eastern Lombardy (AOR: 2.79; 95% CI: 1.01–7.68) and in southern Lombardy (AOR: 2.82; 95% CI: 1.34–5.94) were more frequently infected by SARS-CoV-2. Except the estimates for geographic area, none of the AORs for other socio-demographic characteristics reached statistically significance. However, we found higher AORs in men (compared to women, AOR: 1.64) older subjects (compared to subjects aged <35, AOR: 1.56 for those aged 35–49 years and AOR: 1.48 for ≥50 years), participants living in relatively small municipalities (compared with subjects living in capitals of Lombardy provinces, AOR: 1.27) and administrative personnel (compared to dentists, AOR: 2.18).

None of the AOR estimates on the relationship between selected lifestyle habits and antibody test were statistically significant (Table 3). The AOR for obese compared with normal weight subjects was 1.50). Moreover, higher odds of being SARS-CoV-2 positive subjects were found in non-smokers (for current compared with non-smokers, AOR: 0.69), in current electronic cigarette users (AOR: 1.44), and current HTP users (AOR: 1.57). No specific pattern was evident for alcohol drinking.

No significant relationship has been found between working during the COVID-19 lockdown and SARS-CoV-2 infection, the AOR being 1.12 for participants working occasionally and 1.38 for those working full-time compared to those not working (Table 4). Overall, the prevalence of SARS-CoV-2 infection was 5.0% among asymptomatic subjects (i.e., participants not perceiving to have been infected by SARS-CoV-2), 12.8% among those uncertain about their positivity, 46.4% among symptomatic subjects (i.e., participants perceiving to have been infected by SARS-CoV-2), and 60.0% among participants reporting to have been certainly infected. Compared to asymptomatic subjects, the AOR of SARS-CoV-2 infection for uncertain participants was 3.03 (95% CI: 1.05–8.69), the AOR for symptomatic participants was 17.0 (95% CI: 8.39–34.4), and the AOR for certain to be infected participants was 29.8 (95% CI: 4.47–198). Considering as gold standard the Ab-RDT, the self-reported perception of SARS-CoV-2 infection (negative: asymptomatic and uncertain subjects combined; positive: symptomatic and certain subjects combined) had a sensitivity of 53.7% (95% CI: 39.6%–67.4%) and a specificity of 92.8% (95% CI: 90.0%–95.0%).

## 4. Discussion

To the best of our knowledge, this is the first study attempting to understand the prevalence of SARS-CoV-2-positive subjects in a population of dental staff employees through the use of an Ab-RDT test. Out of almost 500 dental and administrative staff employees from Lombardy, ~10% resulted positive to SARS-CoV-2 antibody test. Of them, 20% were positive to IgM and 96% to IgG (17% to both IgM and IgG). Our results are in line with the prevalence estimates observed on healthcare providers from Lombardy, one of the Italian regions most hit by the COVID-19 pandemic [3,4]. The study by Sandri and colleagues [15], for example, found that 523 out of 3895 (i.e., 13%) healthcare providers in Lombardy were IgG positive subjects. This rate is higher than the global pooled estimate obtained by a systematic review among healthcare workers (pooled seroprevalence of SARS-CoV-2 antibody test: 7%) [14]. Our findings are also comparable with estimates found for the general population of Lombardy region [22,23]

Although we did not find any significant relationship between sex and SARS-CoV-2 positivity, the prevalence of positive subjects was higher among men (14%) than women (9%). This is in apparent contrast with findings from healthcare employees from Lombardy, showing a higher IgG positivity among women (14%) compared to men (11%) [15].

No statistically significant difference has been found with reference to age, in agreement with other studies conducted in Italy and Spain showing no substantial relationship between age and seropositivity among young and middle-aged adults [28,29].

We found significant differences according to geographic area, the prevalence of SARS-CoV-2 positive workers being higher in eastern and southern Lombardy compared to Milan. This is in broad agreement with the geographic differences of seropositivity observed among healthcare providers [15] and COVID-19 incidence in the general population [2], showing systematically higher prevalence of SARS-CoV-2 infection and COVID-19 mortality in eastern Lombardy compared to Milan.

In line with current literature on healthcare providers [28], no statistically significant relationship has been found between profession and SARS-CoV-2 infection. However, higher positivity prevalence has been found among the administrative staff (17%) rather than among dentists (11%) and hygienists (7%), who are those at a more direct contact with patients, thus potentially at higher SARS-Cov-2 risk of infection. This might be related to a higher perceived COVID-19-related risk among dentists [30], inducing them to a higher adherence to a proper use of personal protective equipment [17].

No association has been found between increasing levels of BMI and seropositivity, in agreement with a larger study on healthcare providers from Lombardy [15].

More than one out of five study participants reported to be current smokers. This finding confirms that in Italy smoking prevalence among dentists remains excessively high for a category of healthcare professionals who should set a good example for their patients [31,32]. In this population, we also observe a relatively high use of electronic cigarettes (8%) and HTPs (5%), much higher than the estimates of the general adult Italian population [33,34]. Even alcohol consumption—together with smoking by far the most important avoidable threat for oral health—is greatly consumed by this Italian dental staff as compared to the general adult Italian population [35]. In fact, one-third of participants declared to consume more than three alcoholic beverages per day.

No significant relationship has been observed between current smoking and SARS-CoV-2 infection. However, the prevalence of positive subjects was lower among current smokers (8%) compared to non-smokers (12%). Some other studies, but not all [36,37], found a reduced prevalence of SARS-CoV-2 positive subjects or incidence of COVID-19 among current smokers rather than never or non-smokers [15,38].

To our knowledge, this is the second study quantifying the role of electronic cigarettes on SARS-CoV-2 infection [39] and the first one quantifying the role of HTPs. Our findings on novel products were based on a relatively limited number of users, which impeded us to find statistically significant association. However, we observed a higher prevalence of SARS-CoV-2 positive subjects among electronic cigarette users and HTP users (15% for both products) compared to non-users (11% for both products). These results are in line with a large study from the USA, showing that electronic cigarette use is linked to a substantially increased risk of COVID-19 among adolescents and young adults [39]. These findings claim that new larger studies are urgently needed in order to understand the link between novel tobacco products on SARS-CoV-2 infection and COVID-19 incidence and progression.

In this population, considering as gold standard the Ab-RDT, the self-reported perception of SARS-CoV-2 infection appeared to be a relatively accurate tool to test SARS-CoV-2 seropositivity, the sensitivity being 54% and the specificity 93%.

Limitations of this study include the relatively limited sample size that was unpowered to observe statistically significant associations for selected relatively uncommon risk factors, including electronic cigarettes and HTP use. Although we do not expect major selection biases, the representativeness of our sample to the general population of the dental staff from Lombardy could not be guaranteed. Strengths include the use in this study of the ab-RDT-KHB, a satisfactorily accurate serological test [26].

These findings have public health implications, being this study the first one attempting to evaluate the prevalence of SARS-CoV-2-positive subjects among a population of dental staff employees. This is also among the few studies providing data on the role of selected lifestyle habits, including electronic cigarette use, HTP use, and alcohol drinking, on SARS-CoV-2 seropositivity. Our data suggest that, despite the close physical contact with the patient, dentists are able to protect themselves through the correct use of protection devices and environmental sanitization, prerogatives that have been characterizing dental professional routine for several decades. Accordingly, a recent study showed that out of 356 dentists from northern Italy, all reported a routinely use of the most common protective personal equipment, including facemasks and gloves, before the COVID-19 pandemic [40].

Our study shows a greater prevalence among administrative personnel, likely due to a lower awareness of the risks or a lack of scrupulous management of protective devices. Therefore, correct information/training is essential for the whole dental staff, including dental hygienists, technicians, and assistants/nurses.

The dentist, therefore, seems to be able to deal with the risk of contagion in the workplace, despite the lack of knowledge shown by the recent appearance of SARS-CoV-2, with benefits for the health of the dentists and that of collaborators and patients. However, strict control, proper training of medical and non-medical personnel, functional management of the practice and following guidelines remain of crucial importance. The measures put in place promptly for the management of the SARS-CoV-2 emergency in the dental field, including telephone triage, temperature control, or space management, appear to be effective but further studies are necessary to assess the risk [17].

## 5. Conclusions

In conclusion, although dentistry and related professions have been highlighted at the beginning of the pandemic as high-risk jobs, previous ability to manage diseases with similar transmission mechanisms allowed the dentist to quickly adapt and to continue to provide medical services that guarantee patients’ safety, reducing the risk of infection for the operator.

## Figures and Tables

**Table 1:** Distribution of 499 workers in dental centers from Lombardy region, Italy, by SARS-CoV-2 positivity, overall and by sex and age. Lombardy 2020.

Individual Characteristics	Total No. of Subjects	SARS-CoV-2 Test			
		Negative	IgM+	IgM+/IgG+	IgG+
Total	499	445 (89.2)	2 (0.4)	9 (1.8)	43 (8.6)
Sex					
Men	167	143 (85.6)	0 (0.0)	2 (1.2)	22 (13.2)
Women	332	302 (91.0)	2 (0.6)	7 (2.1)	21 (6.3)
Age group (years)					
<35	163	150 (92.0)	1 (0.6)	2 (1.2)	10 (6.0)
35–49	142	125 (88.0)	1 (0.7)	3 (2.1)	13 (9.2)
≥50	194	170 (87.6)	0 (0.0)	4 (2.1)	20 (10.3)

**Table 2:** Distribution of 499 workers in dentistry with valid test, by SARS-CoV-2 positivity, overall and according to selected socio-demographic characteristics. Corresponding adjusted odds ratios * (AOR) and 95% confidence intervals (CI). Lombardy region (Italy), 2020.

Individual Characteristics	Total No. of Subjects	Subjects Positive to SARS-CoV-2	
		%	AOR (95% CI)
Total	499	10.8	
Sex			
Women	332	9.0	1.00 ^
Men	167	14.4	1.63 (0.91–2.92)
Age group (years)			
<35	163	8.0	1.00 ^
35–49	142	12.0	1.56 (0.73–3.34)
≥50	194	12.4	1.48 (0.72–3.05)
Geographic area °			
Milan	266	9.0	1.00 ^
Northern Lombardy	124	7.3	0.74 (0.33–1.67)
Eastern Lombardy	31	19.4	**2.79 (1.01–7.68)**
Southern Lombardy	65	21.5	**2.82 (1.34–5.94)**
Size of municipality °			
Large	237	10.1	1.00 ^
Small	249	11.6	1.27 (0.71–2.28)
Profession			
Dentists	183	10.9	1.00 ^
Dental technicians	23	13.0	0.95 (0.25–3.59)
Dental hygienists	28	7.1	0.95 (0.19–4.64)
Dental assistants/nurses	179	8.4	1.17 (0.49–2.78)
Administrative personnel	60	16.7	2.18 (0.88–5.38)
Students and others	26	15.4	1.77 (0.54–5.78)

* Estimated by multiple logistic regression models after adjustment for sex and age group. Estimates in bold are those statistically significant at a 0.05 level. ^ Reference category. ° The sum does not add up to the total because of a few missing values.

**Table 3:** Distribution of 499 workers in dentistry with valid test, by SARS-CoV-2 positivity, overall and according to selected lifestyle habits. Corresponding adjusted odds ratios * (AOR) and 95% confidence intervals (CI). Lombardy region (Italy), 2020.

Individual Characteristics	Total No. of Subjects	Subjects Positive to SARS-CoV-2	
		%	AOR (95% CI)
BMI °			
Normal weight	364	10.4	1.00 ^
Overweight	111	10.8	0.75 (0.35–1.59)
Obesity	23	17.4	1.50 (0.48–4.74)
Smoking habit			
Non-smoker	396	11.6	1.00 ^
Current smoker	103	7.8	0.69 (0.31–1.53)
Electronic cigarettes °			
Non-user	470	10.6	1.00 ^
Current user	27	14.8	1.44 (0.47–4.36)
Heated tobacco products °			
Non-user	455	10.5	1.00 ^
Current user	41	14.6	1.57 (0.62–3.97)
Alcohol			
Abstainer	135	10.4	1.00 ^
1–3 drinks/day	183	8.2	0.72 (0.33–1.56)
>3 drinks/day	153	12.4	1.00 (0.46–2.18)

* Estimated by multiple logistic regression models after adjustment for sex and age group. ° The sum does not add up to the total because of a few missing values. ^ Reference category.

**Table 4:** Distribution of 499 workers in dentistry with valid test, by SARS-CoV-2 positivity, overall and according to selected COVID-related characteristics. Corresponding adjusted odds ratios * (AOR) and 95% confidence intervals (CI). Lombardy region (Italy), 2020.

Individual Characteristics	Total No. of Subjects	Subjects Positive to SARS-CoV-2	
		%	AOR (95% CI)
Working during lockdown			
No	243	9.5	1.00 ^
Occasionally	159	11.3	1.12 (0.58–2.16)
Yes	97	13.4	1.38 (0.66–2.89)
Self-reported perception of being SARS-CoV-2 positive			
No	399	5.0	1.00 ^
Uncertain	39	12.8	**3.03 (1.05–8.69)**
Yes	56	46.4	**17.0 (8.39–34.4)**
Yes, certainly	5	60.0	**29.8 (4.47–198)**

* Estimated by multiple logistic regression models after adjustment for sex and age group. Estimates in bold are those statistically significant at a 0.05 level. ^ Reference category.

## Data Availability

Data are available upon reasonable request to the corresponding author.
